# Salt Used for the National School Nutrition Program (NSNP) in Rural Schools of Limpopo Province, South Africa, has Adequate Levels of Iodine

**DOI:** 10.1155/2021/5522575

**Published:** 2021-05-31

**Authors:** Mpho Ramugondo, Lindelani Fhumudzani Mushaphi, Ngoako Solomon Mabapa

**Affiliations:** ^1^Department of Health, Nutrition Section, University of Venda, Mopani District 1391, Phalaborwa, South Africa; ^2^Department of Nutrition, School of Health Sciences, University of Venda, Thohoyandou 0950, South Africa

## Abstract

**Background:**

Salt iodisation is considered the most effective long-term public health intervention for achieving optimal iodine nutrition. Effective salt iodisation is a prerequisite for the sustainable elimination of iodine deficiency disorders. The aim of this study was to determine iodine concentration of salt used for the National School Nutrition Program (NSNP).

**Methods:**

A cross-sectional study was conducted in 359 food handlers from Vhembe and Mopani districts of Limpopo Province, South Africa. The questionnaire was administered to solicit data on demographic information, general questions on salt fortification, and iodine nutrition knowledge. After the interviews, two tablespoons of salt used for the NSNP food preparation was collected from 318 schools in small zip-lock plastic bags. The salt samples were coded and stored at room temperature and protected from light and moisture until the time of analysis. Salt iodine concentrations were determined at the North-West University (NWU) in Potchefstroom by means of the iCheck test method.

**Results:**

The median iodine concentration of both Mopani (31.65 ppm) and Vhembe (32.56 ppm) districts signified adequate iodine levels. Of 318 salt samples, 113 (71%) samples in Mopani and 104 (65%) in Vhembe had an iodine concentration of 15–64 ppm. A few (6%) food handlers in Mopani and almost half (45.9%) in Vhembe could correctly identify iodated salt as the main source of iodine. Almost half of the food handlers (%) in Mopani and 36.5% in Vhembe did not know which part of body needs iodine for functioning.

**Conclusion:**

More than 20 years after the implementation of the USI program, the result of the study shows that the international goal of 90% coverage is still far from being realised.

## 1. Introduction

Malnutrition is a risk factor for early mortality and morbidity in children and adolescents [1]. Malnutrition and hunger accounts for nearly half of the death rate of children worldwide, with approximately 26% of undernourished children residing in Africa [2]. An estimated one-fifth (20%) of the total population comprises those of school age. It is said that approximately 25% of all children in developing countries are vitamin A deficient, whilst other nutrients most likely to be deficient in school aged children are reportedly iron and iodine, with the prevalence rates of the latter being between 35% and 70% [3–7]. Iversen et al. [5] report that undernutrition particularly affects young children residing in rural areas of the country.

Nutrients are not only important for growth and development but also offer children with energy to perform physical and metabolic functions [8]. Nutrition influences the efficiency of educational programmes [9]. Thus, adequate nutrition is vital during the school-age years because nutrition and health influences a child's cognitive development [3, 10]. Children lacking certain nutrients in their diet and who suffer from persistent hunger, parasitic infections, or other nutrition-related diseases are likely to have reduced potential for learning compared to healthy, well-nourished children [11]. Nutrient deficiencies account for the inability of children to achieve their full mental and physical potential because of stunted growth, low physical work capacity, reduced IQ, and a lower resistance to infection [4].

In response to malnutrition, shortly after independence in 1994, the South African government initiated the Primary School Nutrition Programme (PSNP) under the purview of the Department of Health to promote and advocate for malnutrition control, nutritional education, and dietary guidelines in children from low socioeconomic status [5]. The programme was renamed the National School Nutrition Program (NSNP), and implementation was transferred to the Department of Education in 2004 to strengthen action and buy in. The NSNP was introduced as a vehicle for transporting micronutrients such as iron, iodine, and vitamin A to school-aged children [12]. The deficiencies of the three micronutrients of public health significance are iron deficiency anaemia (IDA), iodine deficiency disorders (IDD), and vitamin A deficiency (VAD). Iodine deficiency holds a notorious place among the micronutrient deficiencies which now poses a major worldwide health problem as it is the leading cause of both thyroid and brain abnormalities in children [13].

Salt iodisation is considered the most effective long-term public health intervention for achieving optimal iodine nutrition [14]. Effective salt iodisation is a prerequisite for the sustainable elimination of iodine deficiency disorders. In South Africa, direct and indirect evidence of continued endemic goitre and iodine deficiency led, in 1995, to the introduction of mandatory iodisation of table salt at an iodine concentration of 40–60 ppm. In 2007, the revision of the regulation led to a bigger acceptable range of 35–65 mg iodine/kg salt. Most studies have determined the iodine concentration of household salt but neglected the salt used in the NSNP. This observation underlines the need to determine the iodine concentration of salt used in the NSNP. The main aim of the present study was to determine the iodine concentration of salt used in the NSNP. This may assist in explaining the excessive urinary iodine concentration (UIC) that has been reported recently in South African school children. The secondary aim was to determine the knowledge of food handlers about iodine nutrition and salt iodisation.

## 2. Methods

### 2.1. Sample Design

The study was conducted in selected schools of Mopani and Vhembe districts receiving the NSNP. The Mopani District is situated in the north-eastern part of Limpopo Province. It is the second biggest of the five districts in Limpopo Province. According to the 2016 community survey, it has an estimated population of 1159185 of whom the majority speak Xitsonga and Northern Sotho. There are 16 urban areas (towns and townships), 354 villages (rural settlements), and a total of 125 wards. There are 562 schools on the NSNP in the Mopani District. On the other hand, the Vhembe District is a category C municipality located in the northern part of Limpopo Province. The district has an estimated population of 1294722 of whom the majority speak Tshivenda and Xitsonga, and the settlement pattern is largely rural with approximately 774 dispersed villages [15]. There are 968 schools on the NSP in the Vhembe District.

Simple random sampling was used to select schools that were included in the sample. A list of schools (both secondary and primary) was obtained from the Departments of Education in Vhembe and Mopani districts. The target population was food handlers employed in schools receiving the NSNP from the two districts. Food handlers are persons responsible for preparing meals for school children and preventing outbreaks of food-borne diseases in schools by adhering to food safety guidelines. A total of 359 food handlers (Mopani = 200 vs. Vhembe = 159) were conveniently selected from 318 schools of the two districts. In Mopani, the principals insisted that all food handlers participate in the study, hence an extra 41 food handlers. The two districts were selected based on their history of reported cases iodine deficiency. The study's population was from both rural and urban areas. Food handlers in schools receiving the NSNP in the Mopani and Vhembe districts, 18 years and above, were appointed formally by the school governing body and trained before presuming duties as food handlers and were included in the study. Voluteers and relatives who were standing in for food handlers on the day of data collection were excluded from the study. The reason for the exclusion was the fact that they did not receive training.

### 2.2. Data Collection

Data were collected at the school sites from May 2017 to March 2018. An existing validated researcher-administered questionnaire on knowledge of iodine in the South African adult population [15–17] was used to gather information on iodine nutrition knowledge of food handlers. The questionnaire was administered to solicit data on demographic information, general questions on salt fortification, and iodine nutrition knowledge. The questionnaire was translated into the local languages (Tshivenda, Northern Sotho, and Xitsonga) by the researchers. An expert from the Department of Languages was consulted to translate the questionnaire back to English, and the two were compared. After completing the questionnaire, two tablespoons of salt used for the NSNP food preparation was collected from 318 schools in small plastic zip-lock bags. The salt samples were coded and stored at room temperature and protected from light and moisture until the time of analysis.

The level of knowledge was asssessed using the iodine nutrition knowledge test. The following scores from Umalusi were used to classify the level of knowledge of food handlers: 0–29.9% = poor; 30–49.9% = fair; 50–69.9% = good; 70–89.9% = very good; and 90–100% = excellent (https://www.umalusi.org.za/docs/research/2013/nsc_pass.pdf).

### 2.3. Measurement of Iodine Concentration in Salt by iCheck

Salt iodine concentrations were determined in the iodine laboratory at the North-West University (NWU) in Potchefstroom by means of the iCheck test method [18]. A total of 318 salt samples were analysed representing 100% successfully analysed samples. The analysis was performed in duplicates. The procedure is outlined as follows:

  First step: the salt was diluted with distilled water. The sample per analysis was 1.0 mL, the concentration range was >3 ppm (mg/kg), and the minimum dilution factor was 1 : 3.
  Second step: the sample was injected in the ready-to-use reagent vial, and if the solution changed colour to purple, it indicated that iodine was present.
  Third step: the diluted solution was then analysed using the photometric determination of iodine colorimetric reaction, the units displayed on the iCheck test device were in mg/L, the linear range was set on 1.0–13.0 mg/L, and the time per analysis was <10 minutes.

The iodine concentrations were classified as oniodised (<5 ppm), inadequately iodised (5–14.9 ppm), adequately iodised (15–64.9 ppm), more than adequately iodised (≥65–79.9), and excessively iodised (≥80 ppm) [19].

### 2.4. Statistical Analysis

The data were entered into Microsoft excel spread sheet and exported to the Statistical Package of Social Science (SPSS). The data were analysed using SPSS version 25, and they were expressed as means, standard deviation, median, and interquartile ranges. For categorical data, the percentages and number of samples are presented. To compare differences between Mopani and Vhembe districts, the Mann–Whitney *U* test and independent *t*-tests were used. *p* <  0.05 was considered statistically significant.

## 3. Results

A total of 359 food handlers were recruited and completed the study.  Table 1 shows the age, level of education, marital status, household income, and number of children. The age of the participants ranged from 18 years to 46 years and above. With regard to the level of education, a majority (Mopani = 64% vs. Vhembe = 82.4%) of food handlers had grades 8–12 in both districts. The marital statuses, single and married, had almost equal percentages in both districts. A majority of food handlers from both districts (Mopani = 82.5% vs. Vhembe = 66%) had an income ranging from R1001 to R2000. Almost all food handlers from both districts had children in the school they are working in (Table 1).

Of 359 food handlers, 147 (73.5%) in Mopani and 93 (58.2%) in Vhembe were trained before they were appointed as food handlers. The training period ranged from one day to a week (Table 1). Half of food handlers (50.5%) in Mopani and 40.9% in Vhembe did not know which topics were covered during training.

### 3.1. The Iodine Content of NSNP Salt

A total of 318 salt samples were collected from Mopani and Vhembe districts. The number of salt samples collected from Mopani and Vhembe districts was 159, respectively. The median iodine concentration of both Mopani (31.65 ppm) and Vhembe (32.56 ppm) districts signified adequate iodine levels. There was no significance difference observed between the medians (*p*=0.428) (Table 2).

Of the 318 salt samples, 113 (71%) samples in Mopani and 104 (65%) in Vhembe had an iodine concentration of 15–64 ppm (Table 3). Few samples in Mopani (9%) and Vhembe (11%) were noniodised. Further fewer samples in Mopani (8%) and Vhembe (4%) were excessively iodised (Table 3).

### 3.2. Salt Fortification

Majority of the food handlers in Mopani (67.5%) and Vhembe (78%) were buying salt from supermarkets. Almost all food handlers in Mopani and Vhembe indicated that they never ran out of salt. A total of 30.5% in Mopani and 42.1% in Vhembe stored salt in containers without lids. More than half of the food handlers in Mopani (56%) and Vhembe (51.6%) did not know how to identify iodiated salt (Table 4).

### 3.3. Iodine Nutrition Knowledge

Frequencies of food handlers' answers to the iodine-related questions are summarised in Table 5. A total of 29% in Mopani and almost a third (31.5%) in Vhembe knew that iodine is a micronutrient. A few (6%) food handlers in Mopani and almost half (45.9%) in Vhembe could correctly identify iodated salt as the main source of iodine. Almost half of the food handlers in Mopani and 36.5% in Vhembe did not know which part of the body needed iodine for functioning. A few in Mopani (3.5%) and Vhembe (9.4%) correctly identified brain damage as the most harmful effect on the health of children if they do not get enough iodine. Half in Mopani (50.5%) and almost half in Vhembe (47.2%) knew the daily recommendation of iodine for school-attending children (Table 5).

The iodine nutrition knowledge of food handlers was poor with majority scores ranging from 0 to 29.9%. The distribution of scores by food handlers is summarised in Table 6.

## 4. Discussion

An iodine concentration of 15–64.9 ppm denotes that the salt is adequately iodised [18]. The results suggest that a majority of the salt samples in Mopani and Vhembe districts were adequately iodised.

A portion of samples was inadequately or noniodised or excessively iodised in the current study. One would expect all salt samples used for the NSNP to be adequately iodised; however, this was not the case in the current study. Inadequate iodine intake may result in a variety of disorders, termed iodine deficiency disorders (IDD), such as goitre, cretinism, spontaneous abortion, perinatal mortality, and heart failure [18, 20–22]. On the other hand, excess iodine may impair thyroid function [23]. Recently, a study [24] conducted in Limpopo Province has reported excess UIC in SAC which is a problem. A possible explanation for the variation in iodine concentration levels could be that, in South Africa, there is no proper monitoring of salt fortifications at production sites [25]. This may result in some salt producers underiodising and some overiodising as observed in the current study.

The use of adequately iodised salt for the NSNP in Mopani (71%) and Vhembe (65%) was below the international coverage of 90%. It is noteworthy to assume that salt producers iodising salt at a concentration of more than 20 ppm contributes to the elimination of iodine deficiency [22].

The results of the current study indicated that food handlers in Mopani and Vhembe stored salt either in the plastic bag in which the salt was bought in or in containers with/without a lid. When iodised salt is not stored in closed plastic bags, sealed waterproof materials, or closed containers, iodine losses occur leading to reduction in the iodine content of salt before it is consumed [26].

Again, when improperly packed iodated salt is transported over long distances under humid conditions, it will attract moisture and become wet, dissolving and carrying the iodate to the bottom of the bag, and finally, it can be lost if the bag is porous to water [27, 28]. Salt packed in such materials may lose as much as 75% of its iodine content over nine months. High-density polyethylene bags and polyethylene laminated bags are recommended for bulk packaging purposes [29]. Contrary to the results of the current study are the findings of a study conducted in Ethiopia where it was reported that most of the salt containers had covers at the household and the majority of caterers stored salt in a cool, dry area [30]. The iodine content of the salt remained constant, and its distribution remained uniform for many months when the salt is packed and kept dry, preferably in a cool place and away from sunlight [31]. Gebremariam et al. [32] also found that using packed salt at the household level was significantly associated with the availability of adequately iodised salt.

The results of the study suggest that there is limited iodine nutrition knowledge among the food handlers working for the NSNP, despite international efforts to eradicate IDD worldwide and the notable progress South Africa and other countries have made towards this goal. It can be concluded that these efforts have not yielded the expected growth regarding the iodine nutrition knowledge. The results of the current study have yielded similar results to studies conducted among the South African adult population [33], where it was found that the knowledge level of iodine nutrition is low, particularly among the low socioeconomic groups. The current study and studies conducted in the Bargarh District [34], Kashmir Valley [35] of India, and the Andaman and Nicobar Islands have demonstrated low levels of iodine nutrition knowledge among the general public. Low levels of iodine nutrition knowledge is evident in the current study since more than a third of participants in Mopani and almost half of those in Vhembe did not know what iodine is, a few knew that salt was the main source of iodine, and half did not know which part of the body required iodine for functioning. Although international efforts have been put in place to emphasise the consequences of iodine deficiency on brain damage, it is surprising to note that the food handlers were not aware that mental retardation may result from iodine deficiency. To close this IDD communication gap, which appears to inhibit the transfer of this message to the consumer level, Jooste Joubert [33] maintains that both educational and public health communication strategies are required. In Turkey, the percentage of women using iodised salt increased significantly during a 3-month regional educational mass media campaign [36], indicating that improved IDD knowledge may lead to a more widespread consumption of iodised salt [33].

Nutrition knowledge is the knowledge of nutrients and foods [37]. Good nutrition knowledge is a rudiment for healthy living. Therefore, adequate nutrition knowledge is needed when purchasing and preparing balanced meals [38]. Apart from purchasing and preparing meals, nutrition knowledge is extremely useful in an intervention programme [39]. However, poor nutrition knowledge affects food choices and dietary interventions and can possibly compromise [40] the purpose of the NSNP. A food handler or cook working for the NSNP needs to know the various nutrient contents of each food type, proper application derived from great ideas, and benefit maximisation [38]. Failure to acquire potential and competent nutrition knowledge by food handlers of the NSNP may jeopardise addressing the micronutrient deficiencies, which is one of the crucial aims of the NSNP that has been widely neglected [41].

## 5. Conclusions

Since the introduction of mandatory salt iodisation by 40–60 ppm in South Africa in 1995, substantial progress has been made to eliminate IDD. More than 20 years after the implementation of the USI program, the results of the study show that the international goal of 90% coverage is still far from being realised. In the IDD Newsletter [42] of November 2013, Jooste indicated that household coverage of adequately iodised salt in South Africa was greater than 77%. Proper measures must be put in place to monitor fortification of salt at production sites. The results also show that food handlers have limited information about the iodine nutrition. Effective iodine nutrition education should be included in the training of food handlers.

## Figures and Tables

**Table 1:** Sociodemographic information of participants.

Sociodemographic information	Mopani district		Vhembe district	
	N	%	N	%
*Age*				
18–25	6	3	3	1.9
26–35	60	30	52	32.7
36–45	82	41	68	42.8
46 and above	52	26	36	22.6

*Level of education*				
Never attended	13	6.5	6	3.8
Grade 1–7	51	25.5	14	8.8
Grade 8–12	128	64	131	82.4
College/tertiary	8	4	8	5

*Marital status*				
Single	88	44	66	41.5
Married	96	48	69	43.4
Divorced	4	2	11	6.9
Living with partner	12	6	13	8.2

*Household income*				
Less than R1000	3	1.5	3	1.9
R1001-R2000	165	82.5	105	66
R2001-R3000	31	15.5	40	25.2
R3001-R4000	0	0	6	3.8
R4001-R5000	1	0.5	5	3.1

*Do you have the child at this school you are working?*				
Yes	188	94	152	95.6
No	12	6	7	4.4

*How many children do you have?*				
None	7	3.5	3	1.9
1-2	58	29	48	30.2
3-4	89	44.5	76	47.8
5 or more	46	23	32	20.1

*Were you given nutrition training before working as a food handler?*				
Yes	147	73.5	93	58.2
No	53	26.5	66	41.8

*How long was the training?*				
1 day	47	23.5	69	43.4
2 days	13	6.5	12	7.5
3 days	11	5.5	1	0.6
A week	54	27	9	5.7
Other	24	12	3	1.9
Do not know	51	25.5	65	40.9

*Topic covered during training*				
Food handling	93	46.5	55	34.6
Food safety and hygiene	5	2.5	37	23
Nutrition	1	0.5	1	0.6
Other	0	0	1	0.6
Do not know	101	50.5	65	40.9

**Table 2:** Iodine content mean values for Mopani and Vhembe districts.

District	Mean ± SD (*N* = 159)	Median (*N* = 159)	*p* value
Mopani	36.53 ± 27.88 ppm	31.65 ppm (IQR: 23.50–43.30 ppm)	0.428
Vhembe	34.24 ± 23.46 ppm	32.56 ppm (IQR:14.41–51.51 ppm)	

**Table 3:** Distribution of salt by iodine concentration.

Iodine content categories	Mopani district		Vhembe district	
	*N*	%	*N*	%
<5 ppm (noniodised)	14	9	18	11
5–14 ppm (inadequately iodised)	13	8	25	16
15–64 ppm (adequately iodised)	113	71	104	65
65–79.9 ppm (more than adequately iodised)	7	4	6	4
≥80 ppm (excessively iodised)	12	8	6	4
Total	159	100	159	100

**Table 4:** General questions on the salt fortification.

Description of questions	Mopani District		Vhembe District	
	*N*	%	*N*	%
*Where do you usually buy or obtain the salt that is used for food in your house?*				
Purchase in a shop such as Pick ‘n Pay, Shoprite, Spar, and general store	135	67.5	124	78
Agricultural coarse salt obtained from a farmer, employer, cooperation, or elsewhere	2	1	1	0.6
Spaza shop	59	29.5	18	11.3
Informal sector: street vendor or hawker and a bag of maize meal	4	2	14	8.2
Directly from a salt producer	0	0	2	1.3

*Where do you get salt if the salt used at school ran out before the service provider delivers the next batch?*				
Buy at a nearby shop/Spaza	15	7.5	3	1.9
Fetch salt at home	1	0.5	1	0.6
Do not add salt	2	1	1	0.6
Never run out of salt	182	91	15	96.9

*Do you add more salt to your food because the salt is iodated?*				
Yes	13	6.5	0	0
No	122	61	67	42.1
Do not know	6	3	3	1.9
Do not know what iodated salt is	59	29.5	89	56

*Do you have any concerns about iodine being added to table salt?*				
Yes	17	8.5	3	1.9
No	107	53.5	50	31.4
Unsure	13	6.5	20	12.6
Do not know what iodine is	63	31.5	86	54.1

*In what kind of container do you store salt in the kitchen?*				
Plastic bag in which the salt was bought	82	41	56	35.2
Carton box	7	3.5	25	15.7
Rigid plastic container with holes at the top	50	25	11	6.9
Open porcelain, wooden, plastic, or metal container with or without a lid	61	30.5	67	42.1

*How do you know the salt is Iodised?*				
Plastic bag/container with iodised salt written on it	50	25	47	29.6
Salt having brown colour	4	2	3	1.9
Salt with pure white colour	34	17	27	17
Do not know	112	56	82	51.6

*When do you add iodised salt to food?*				
While cooking	101	50.5	71	44.6
Start cooking with salt	90	45	88	55.3
While eating	3	1.5	0	0
Do not know	6	3	0	0

**Table 5:** Iodine nutrition knowledge test.

Description of questions	Mopani District		Vhembe District	
	*N*	%	*N*	%
*What is iodine?*				
Vitamin	65	32.5	19	11.9
^*∗*^Micronutrient/mineral	58	29	50	31.5
Something in the food that we eat	4	2	13	8.2
Other (medicine, potion, etc.)	2	1	0	0
Do not know	71	35.5	77	48.4

*Main source of iodine*				
^*∗*^Iodised salt/iodated salt	12	6	73	45.9
Fish/sea food/marine food products	60	30	29	18.2
Vegetables	25	12.5	12	7.5
Meat or meat products	25	12.5	6	3.7
Dairy products	5	2.5	4	2.5
Drinking water	13	6.5	3	1.5
Other	22	11	1	0.6
Do not know	14	7	11	6.9
Do not know what iodine is	24	12	20	12.5

*Part of the body that needs iodine*				
Liver	42	21	47	29.6
^*∗*^Thyroid gland/gland in the front of the neck	40	20	24	15.1
Lungs	24	12	30	18.9
Do not know	94	47	58	36.5

*Most harmful effect on health of children if they do not get enough iodine*				
Slow growth	59	29.5	59	37.1
Goitre/enlarged thyroid gland/swollen neck	22	11	22	13.8
^*∗*^Brain damage or underdevelopment of the brain/low intelligence	7	3.5	17	9.4
Cretinism	2	1	1	0.6
Hypothyroidism	0	0	1	0.6
Death	1	0.5	1	0.6
Diabetes mellitus	7	3.5	4	2.5
Do not know	74	37	29	18.2
Do not know what is iodine	26	13	25	15.7
Hypertension	2	1	2	1.3

*Do you read labelling on the food package to check salt content?*				
^*∗*^Yes	56	28	48	30.2
No	108	54	85	53.5
Cannot read	12	6	2	1.3
Do not know what iodine is	24	12	24	15.1

*What is the daily recommendation of iodine for school-attending children?*				
^*∗*^120 *µ*g/L	101	50.5	75	47.2
150 *µ*g/L	18	9	14	8.8
220 *µ*g/L	2	15	2	1.3
Do not know	79	39.5	68	42.8

^*∗*^ Correct response.

**Table 6:** Iodine nutrition knowledge scores.

Scores (%)	Interpretation	Mopani district		Vhembe district	
		*N*	%	*N*	%
0–29.9	Poor	95	47.5	54	34
30–49.9	Fair	45	22.5	45	28.3
50–69.9	Good	54	27	49	30.8
70–89.9	Very good	5	2.5	8	5
90–100	Excellent	1	0.5	3	1.90

## Data Availability

Data will be available on request.

## Abbreviations

ICCIDD: International Council for Control of Iodine Deficiency Disorders
IDD: Iodine deficiency disorders
MI: Micronutrient initiative
NSNP: National School Nutrition Program
SAC: School-age children
UNICEF: United Nations Children's Fund
USI: Universal Salt Iodisation
WHO: World Health Organisation.
